# Prostatic metastasis of INI-1-deficient Sinonasal carcinoma: the first case report of this exceptional finding

**DOI:** 10.1093/omcr/omag044

**Published:** 2026-05-24

**Authors:** Sabrine Derqaoui, Taha Yassine Aaboudech, Siham Mesmoudi, Ahmed Jahid, Zakia Bernoussi, Kaoutar Znati

**Affiliations:** Department of Pathology, Ibn Sina University Hospital Center, abbderrahim avenue, rabat postal 12000, Rabat, Morocco; Faculty of Medecine and Pharmacy, Mohamed V University Rabat, abbderrahim avenue, rabat postal 12000, Morocco; Department of Pathology, Ibn Sina University Hospital Center, abbderrahim avenue, rabat postal 12000, Rabat, Morocco; Faculty of Medecine and Pharmacy, Mohamed V University Rabat, abbderrahim avenue, rabat postal 12000, Morocco; Department of Pathology, Ibn Sina University Hospital Center, abbderrahim avenue, rabat postal 12000, Rabat, Morocco; Faculty of Medecine and Pharmacy, Mohamed V University Rabat, abbderrahim avenue, rabat postal 12000, Morocco; Department of Pathology, Ibn Sina University Hospital Center, abbderrahim avenue, rabat postal 12000, Rabat, Morocco; Faculty of Medecine and Pharmacy, Mohamed V University Rabat, abbderrahim avenue, rabat postal 12000, Morocco; Department of Pathology, Ibn Sina University Hospital Center, abbderrahim avenue, rabat postal 12000, Rabat, Morocco; Faculty of Medecine and Pharmacy, Mohamed V University Rabat, abbderrahim avenue, rabat postal 12000, Morocco; Department of Pathology, Ibn Sina University Hospital Center, abbderrahim avenue, rabat postal 12000, Rabat, Morocco; Faculty of Medecine and Pharmacy, Mohamed V University Rabat, abbderrahim avenue, rabat postal 12000, Morocco

**Keywords:** Audiovestibular medicine, critical care medicine

SMARCB1 or INI-1 deficient carcinoma, is a rare sinonasal malignant epithelial tumor, accounting for 1%–3% of all sinonasal malignant neoplasms [1]. Given its aggressiveness and fatal outcome, SDSC (SMARCB1 deficient sinonasal carcinoma) should be distinguished from other undifferentiated sinonasal carcinoma, and further immunophenotypic study and/or molecular work-up must be performed [2]. SDSC is defined as undifferentiated carcinoma lacking squamous or glandular differentiation with complete loss of SMARCB1/INI1 expression and SMARCB1 mutation+/−SMARCA2 mutation upon genetic studies. However, molecular testing is not mandatory for the diagnosis [3]. Histologically, SDSC usually presents as undifferentiated carcinoma with basaloid features lacking glandular or squamous differentiation [4]. On immunohistochemistry, the neoplastic cells express pan-cytokeratin, with focal expression of p63/p40. NUT and p16 are negative. The clue of diagnosis is INI 1 expression’s loss [3]. Because of its high aggressiveness and poor prognosis, patients usually present at a locally advanced or metastatic disease [4]. Most common metastatic sites reported to date are: leptomenigeal, lung, bone and liver. However, prostatic metastasis have never been previously reported [5]. Herein we describe a case of 70- year-old man, with locally advanced SDSC presenting with distant metastasis to prostate during the follow up period. To the best of our knowledge this is the first case of prostatic metastasis of SDSC.

Our patient, presented with a locally advanced SDSC (Fig. 1A and B), treated by chemotherapy in association to radiotherapy. During a follow up period of six months, the patient developed urinary obstructive syndrom, without serum elevated PSA. A transurethral prostatic resection was performed. Upon histological examination, the prostatic parenchyma was infiltrated by solid nests of basaloid cells (Fig. 1C and D). The neoplastic cells were positive for P40 and P63 (Fig. 1 E) with complete loss of INI1 (Fig. 1F). S100, p16, PSA (Fig. 2A) and NUT were negative. Pan cytokeratines were positive (Fig. 2B). Based on morphological and phenotypical features, supported by previous history, the diagnosis of SNSC metastasis to prostate was retained. The patient had fatal outcome, and died 1 month later, in the setting of pulmonary emboli.

**Figure 1:**
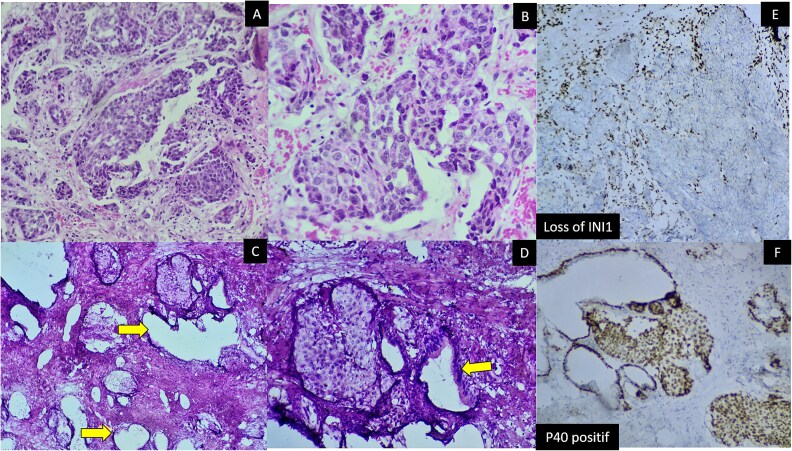
(A) Sinonasal lesion’s histological findings (HE medium power). (B) Sinonasal lesion’s histological findings (HE high power). (C) Transurethral prostatic resection findings: Prostatic glands (yellow arrow) adjacent to carcinomatous solide nests. (HE medium power). (D) Transurethral prostatic resection findings: Prostatic glands (yellow arrow) adjacent to carcinomatous solide nests. (HE power high). (E) Immunohistochemistry, INI1 loss (prostatic tumor). (F) Immunohistochemistry P40 positive in carcinomatous solid nests with positivity in basal cells of normal adjacent prostatic glands. (prostatic tumor).

**Figure 2:**
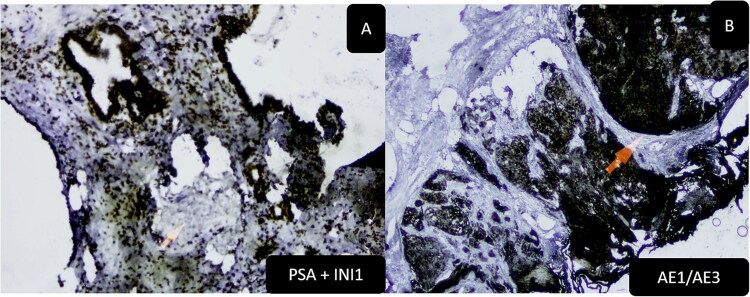
(A) Immunohistochemistry, PSA loss, with positivity in prostatic glands (PSA stain with INI1 stain). (B) Immunohistochemistry AE1/AE3 positive in carcinomatous solid nests with positivity in normal adjacent prostatic glands.

In conclusion, SDSC is a highly aggressive tumor, with poor outcome, which should be considered in the diagnosis of sinonasal undifferentiated carcinoma. Prostatic metastasis is an exceptional presentation. Further studies are needed to elucidate its mechanism and to treatment outcomes and survival.

## Data Availability

Not applicable.
